# Moral psychology from the lab to the wild: Relief registries as a paradigm for studying real-world altruism

**DOI:** 10.1371/journal.pone.0269469

**Published:** 2022-06-13

**Authors:** Brendan Bo O’Connor, Karen Lee, Dylan Campbell, Liane Young

**Affiliations:** 1 Department of Psychology, University at Albany, State University of New York, Albany, New York, United States of America; 2 Department of Psychology, Boston College, Chestnut Hill, Massachusetts, United States of America; Heidelberg University, GERMANY

## Abstract

Experimental psychology’s recent shift toward low-effort, high-volume methods (e.g., self-reports, online studies) and away from the more effortful study of naturalistic behavior raises concerns about the ecological validity of findings from these fields, concerns that have become particularly apparent in the field of moral psychology. To help address these concerns, we introduce a method allowing researchers to investigate an important, widespread form of altruistic behavior–charitable donations–in a manner balancing competing concerns about internal validity, ecological validity, and ease of implementation: relief registries, which leverage existing online gift registry platforms to allow research subjects to choose among highly needed donation items to ship directly to charitable organizations. Here, we demonstrate the use of relief registries in two experiments exploring the ecological validity of the finding from our own research that people are more willing to help others after having imagined themselves doing so. In this way, we sought to provide a blueprint for researchers seeking to enhance the ecological validity of their own research in a narrow sense (i.e., by using the relief registry method we introduce) and in broader terms by adapting methods that take advantage of modern technology to directly impact others’ lives outside the lab.

## Introduction

The methods used in the typical social psychology study have substantially changed in recent years. Studies published in top journals in the field are more frequently conducted online, are shorter in duration, and rely more heavily on self-report measures as compared to studies published in these same journals 15 years ago [1, 2]. This shift towards online, low-cost, high-volume methods is believed to have resulted from a combination of factors including calls for greater statistical power and replicability in the field [2], incentives for publication and competition over limited resources in academia [1, 3], and a prioritization of internal over external validity in decisions about what sorts of research to publish [4]. The trend toward online studies in particular has likely been accelerated by the heightened need to conduct research remotely brought on by the COVID-19 pandemic. Meanwhile, more effortful field research involving the study of cognition and behavior in naturalistic settings [e.g., 5] has become increasingly scarce in psychology, creating concerns about whether results obtained in the lab or online would generalize to real-world settings [3, 4, 6–12]; in other words, whether these findings are ecologically valid in the sense that this term has recently been used in experimental psychology [9, 13–16]. These competing concerns and practical constraints create a clear need for realistic behavioral measures and manipulations that would facilitate higher levels of ecological validity without compromising ease of implementation or internal validity.

Perhaps nowhere has this need been felt more acutely than in the field of moral psychology, which has traditionally relied heavily on vignettes, questionnaires, games, and thought experiments such as the trolley problem as opposed to the observation of moral behavior itself [e.g., 17–26]. These approaches are often highly appropriate, such as when researchers are primarily interested in unobservable cognitive phenomena (e.g., judgments of right and wrong) or when ethical concerns preclude the naturalistic study of the behavior of interest (e.g., decisions in life-or-death moral dilemmas) [22, 27]. Yet a growing number of studies make clear that procedures relying solely on hypothetical situations and self-reports about behavior often fail to predict moral behavior in more realistic settings. Clear gaps between intentions and behavior have been shown in domains including responses to racism and sexual harassment [28, 29], the desire for retributive punishment [30], willingness to forego personal gain to prevent harm to others [31], interventions in moral violations such as the theft of personal property [32], judgments in sacrificial harm dilemmas like the trolley problem [33], compliance with unethical requests made by authority figures [34], and of particular relevance to the current research, monetary donations [35]. Thus, what participants say with regard to morality is sometimes a poor guide to what they actually do, suggesting that moral psychologists in particular should be attentive to the extent to which the experimental procedures they use are representative of the naturalistic settings in which the behaviors of interest unfold [36, 37].

One of the most heavily studied forms of moral behavior in psychology and behavioral economics is the allocation of resources in the form of charitable donations, no doubt due to the ubiquity and importance of this particular form of prosociality. Indeed, American citizens alone currently donate over 400 billion dollars to charity each year [38]. Researchers have operationalized charitable donations by a variety of means, including the use of preexisting data sets on real-world giving [e.g., 39, 40], reports about past donation behavior [41], self-reported intentions or attitudes related to donating [42, 43], economic games [44], and actual donations made in laboratory or field studies [45, 46]. Decisions about which of these measures to use often involve tradeoffs between various methodological concerns. For example, measuring hypothetical donations in a lab setting allows for greater ease of use and internal validity but may be lacking in the real stakes and rich contextual details present in real-world donation decisions [47]. On the other hand, exploring real-world donations might mean relying on preexisting data sets that preclude causal inference or field studies where internal validity and ease of data collection are more variable.

Researchers in psychology and economics have been aware of these issues for some time [4, 6, 48, 49] and the “gold standard” approach for addressing them has been the field experiment, in which an experimental manipulation is embedded directly into a real-world charitable appeal, reconciling concerns between internal and external validity [e.g., 50–52]. Such studies remain relatively rare in social psychology, however, where self-report and hypothetical measures are still the norm [6, 7, 53]. The availability of a wider variety of ecologically valid measures of charitable giving–particularly those bridging methods psychologists are comfortable and familiar with at this point (e.g., online survey studies) [2] with those typically employed in field experiments–may thus be of value to psychology, a field in which concerns about the generalizability of findings to real-world settings is widespread [3, 4, 6, 7, 12].

To this end, the current research introduces a method which reflects the expanding application of technology to helping others in need: the use of online platforms originally designed for the creation of gift registries to allow participants in the lab or online to donate needed items to real-world charitable organizations, which we created and refer to here as a relief registry. Whereas charitable donations are highly common [38], charitable giving can often be inefficient and wasteful due to factors such as a lack of information about the available donation options and their relative efficacy [54, 55], and the personal preferences and social biases that influence donation decisions [56, 57]. As a result, well-intentioned donors may nonetheless end up donating items that are not actually needed [58, 59], as has been the case in the aftermath of recent disasters including Hurricane Sandy [60, 61] and the 2004 Indian Ocean tsunami [62]. Relief registries resolve these issues by connecting charitable organizations directly with donors, providing an easy-to-use mechanism by which high-priority donation items can be shipped directly to the places and people in the greatest need, and in the current context, allowing psychological research to have immediate, real-world impact. Indeed, similar relief registries to those used in the current research were successfully implemented in the aftermath of Hurricane Sandy [60–62].

These relief registries possess a number of desirable properties: They can be easily implemented in the laboratory or online, including alongside experimental manipulations and sampling procedures allowing for high levels of internal validity, while retaining the essential features of realistic donations in terms of overt characteristics (as charitable giving increasingly occurs online) [63] and the realistic psychological stakes involved, as participants are asked to give up real resources with immediate, tangible prosocial consequences. In this way, we see relief registries as overlapping with and complementary to standard field experimental approaches to charitable giving while modifying and building off of these approaches in several ways which may make them more useful or accessible within certain research contexts. First, relief registries were created to be relatively easy to incorporate into the sorts of studies which have become increasingly common in psychology (i.e., survey studies conducted online [2]) and thus, may be more readily accessible for many researchers as compared to “true” field experiments (i.e., experimental manipulations carried out in naturalistic settings) which often require a greater amount of effort, planning, and coordination to successfully carry out [3]. Thus, relief registries offer a possible alternative for researchers wishing to investigate charitable giving online rather than in person and in this way, complement similar research in experimental economics which has investigated naturalistic giving in online samples (e.g., [64]). The fact that relief registries operate via the exchange of needed goods rather than monetary donations also sets them apart from much preexisting research [e.g., 46, 65, 66], broadening the sorts of experimental designs in which they can be used (e.g., the two original experiments described below, which rely on the donation of specific items rather than money). Further, the clear link between participants’ actions (i.e., the selection of donation items in the relief registry) and prosocial outcomes (i.e., these same items being sent directly to charities) may yield greater psychological benefits for participants as compared to studies relying on monetary donations, which involve further intermediary decisions about how to spend said money occurring outside of participants’ awareness. In this way, the current research complements a growing literature within experimental economics focused on how framing donation requests in terms of tangible goods vs. money impacts total donation amounts, individuals’ propensity to give, and their motivations when giving (e.g., [64, 67, 68]). For these reasons, we see relief registries as a useful addition to the methodological toolbox available to those wishing to heighten the ecological validity of their research while acknowledging that standard field experimental approaches may still be preferable in many cases.

To demonstrate the use of relief registries in action, we coordinated efforts with non-profit organizations doing a variety of charitable work in the Greater Boston area, asking these organizations to provide a list of objects and items that they needed to best serve their community and help people in need. We then uploaded these objects onto an adapted wedding registry site (myregistry.com), used images of these objects as stimuli and provided subjects the opportunity to donate these items using the registry. Donated items could then be directly shipped to the places and people that need them the most. We conducted two preliminary experiments demonstrating the use of relief registries to test the external validity of a recent finding from our labs that people are more likely to help others in need after they have imagined themselves doing so [20, 69] and that the vividness and detail of imagined scenes partially mediates this relationship [70]. Whereas prior research testing these hypotheses has relied primarily on a measure of prosocial intention (i.e., participants’ self-reported willingness to help a target individual in need) [20, 71] or donations to hypothetical individuals [70], we tested whether imagination would exert similar effects on people’s actual donation behaviors when using relief registries. Specifically, we tested whether people would be more likely to donate items they had imagined themselves using to help others as compared to conditions controlling for exposure to donation items and descriptions of others in need (Study 1), and whether manipulating the strength of the visuospatial context (via the familiarity of the location where imagined events took place) causally affected the strength of this relationship (Study 2).

## Study 1

### Method

All studies presented here were approved by the Boston College Institutional Review Board and all participants provided informed consent prior to taking part in these studies. Data for the current studies was initially collected in 2015–2016. We note that our main intention in the current research was to provide readers with a practical demonstration of the use of relief registries rather than make strong claims regarding the specific hypotheses we test here. Particularly due to the relatively small sample sizes used in both studies, readers should be cautious not to overinterpret the specific effects presented below. We note that, whereas both labs involved in this research currently use and encourage pre-registration, this data was collected prior to these labs adopting policies of routine pre-registration of hypotheses and procedures.

#### Participants

Thirty-six undergraduates from Boston College and Boston University were recruited to participate in a study on reactions to stories from various media sources and were paid $25 for their participation with an additional $20 bonus (see details in the description of the relief registry task below). Five participants were excluded from data analysis for donating all of the items on the relief registry. This was done to address a minor design flaw in this study, as allowing participants to simply donate one of each item does not provide any useful information about variance across conditions when subjects are forced to choose between and prioritize different donation items. However, we note that including these subjects in our analyses did not affect our results and we replicated these findings in Study 2 after fixing this issue in the design itself. Additionally, one participant was excluded for not complying with task instructions on six or more (i.e., more than 20%) of the 30 trials in the imagined helping and control tasks described below. The final sample consisted of *N* = 30 individuals (age 18–27 years, *M* = 19.63 years, *SD* = 1.83, five males). A power analysis indicated that this sample size would allow for the detection of an effect of *d*_*Z*_ = 1.32 (corresponding to the standardized mean difference in willingness to help between Imagined Helping and control trials found in prior work) [20] in nearly all cases (i.e., calculated power rounded to 100%). Despite this power analysis, we again acknowledge that the sample sizes in the two current studies are fairly small and thus, some caution is warranted in interpreting the inferential statistical tests presented below.

#### Procedure and materials

*Stimuli*. We contacted seven non-profit organizations from the Boston area (e.g., Boston Natural Areas Network, Centre Street Food Pantry) to create a list of 40 items that were highly needed for donations. We then created 40 different stories describing anonymous individuals in need, each of which corresponded to one item from this list (i.e., scenarios in which the person described could be helped using the item in question). For example, for “umbrella”, the corresponding story was, “An elderly person is walking home with their hands full of groceries and it starts to rain.” Images of each item were obtained from the internet and paired with these stories to create 40 unique story/image pairs for use in the study, all of which are available at https://osf.io/ru74z/?view_only=402ebd165a1843838c7a21496e80d597. Finally, we created a relief registry modeled after wedding/baby gift registries using the website myregistry.com. This registry consisted of all 40 highly needed donation items which could be purchased through Walmart or Amazon.com.

*Imagined helping and control tasks*. Upon providing informed consent, participants completed 30 trials of a task adapted from prior work [e.g., 20, 69, 70, 72] that involved the story/image pairs described above. Instructions on these trials (i.e., what participants were told to do in relation to each story/item pair) were manipulated within-subjects (Condition: Imagine vs. Story vs. Object). On 10 of these trials, participants saw a story/image pair randomly selected from the full set of 40 and were instructed to imagine themselves helping the individual described using the pictured item at a specific time and place (Imagine condition). On another 10 of these trials, participants were instructed to think about the writing style of the story and based on this, what sort of media source it likely came from (e.g., newspaper article, blog, social media post; Story condition). On the remaining 10 trials, participants were shown only images of donation items without accompanying stories and were instructed to design imaginary advertisements for these items in any medium, considering things like the text, color, and format of these advertisements as they visualized them (Object condition). The Story condition served to control for exposure to, and consideration of, the descriptions of people in need. The Object condition controlled for exposure to (and thus, possible priming or familiarity effects of) the picture of the item that subsequently appeared on the relief registry at the end of the study. On each trial, participants viewed the stimulus (i.e., either the story/image pair or the image alone) for 10 seconds and then performed the assigned task for that trial for 60 seconds. The assignment of each story/item pair to condition was randomized for each participant, as was the order in which stories appeared. Thus, participants saw a random selection of 30 of the full set of 40 donation items during this task.

Before completing the experimental trials, participants received verbal instructions and visual examples of a trial for each of the three conditions, then completed two practice trials per condition to ensure task comprehension. Experimenters provided feedback during these practice trials and addressed any questions as needed. Participants were asked to play close attention to the tasks and were told that they would be answering follow-up questions about these tasks later in the study. Participants also wrote out brief descriptions of their imagined events (Imagine condition), the writing style and media source they identified (Story condition), or the advertisement they imagined (Object condition), which were used to evaluate task compliance for the data exclusions described above.

*Dependent measures*. Self-report. After the completion of all 30 trials, the 30 story/image pairs were re-presented to participants in the same order as in the previous portion of the study (we note that the stories for the Object condition were new to participants, as they had only seen images in the previous part of the study). Participants completed a self-paced survey consisting of the following items pertaining to each story/image pair (although due to the nature of some items, they were only answered for certain conditions): willingness to help (“How likely would you be to help in this situation?”, from 1 = not at all to 7 = very willing; Imagine and Story conditions only), affective valence (“How emotionally positive or negative were the events you imagined, the journalistic techniques you identified, or the advertisements you designed?”, from 1 = highly negative to 7 = highly positive; all three conditions), the coherence and scene detail of imagined helping episodes or advertisements (“The imagined scene in your mind was…”, from 1 = vague to 7 = clear and coherent and 1 = simple to 7 = detailed; Imagine and Object trials only) and one item (preliving) assessing the sensation of mentally experiencing the event as though it were currently occurring (“How strongly did you experience the imagined event in your mind?”, from 1 = not at all to 7 = vividly, as if I were there; Imagine trials only). Following prior work [20], scene detail and coherence were combined into a single index (Cronbach’s α = .93) which we refer to below as vividness.

Relief registry. At the completion of the survey, participants learned that they would receive an additional $20 endowment on top of the $25 payment they were to receive for their participation. Participants were told that they could use this $20 to donate specific items to charity using an online registry prepared for the purposes of this study. Participants were then linked to the relief registry described above. This registry consisted of all 40 items highly needed for donations: 30 of which they had seen during the imagined helping and control tasks, and 10 of which were unfamiliar to them (New condition). All items were accompanied by the images mentioned in the ‘Stimuli’ section above, though no additional information about these items was included.

Participants were told that they could use as much of the $20 endowment as they liked to donate items from the registry to local charities in the Boston area and that these items would be directly donated to individuals in need. Each item cost $0.50 and the remainder of the cost of each item was to be covered by the research lab, but participants were told that they were under no obligation to donate items and could keep any amount of money that they chose not to donate. Participants then checked boxes next to items on the registry they wished to donate; participants could select as many different donation items as they wished but were limited to a quantity of one per item. All selected items were shipped directly to the charitable organizations mentioned above and multiple items were shipped when multiple participants selected the same item. Participants remained unaware of the identity of the specific charities involved until the conclusion of the study. At the close of the study, participants were then debriefed and thanked for their time.

### Results

Data for both studies is available at https://osf.io/c56wr/?view_only=ae3964a08ea249938a3089f6d299a42f. We used generalized linear mixed-effects regression analysis to test for condition effects on our focal dependent measures while treating both participants and stimuli (i.e., story/image pairs) as random factors (i.e., including both random intercepts and slopes for these variables). This analysis assumes that both participants and stimuli reflect random samples from larger populations and avoids the biased estimates and inflated type I error which result from ignoring natural variation across stimuli [73]. We found an effect of imagined helping on prosocial intentions: willingness to help the target in need was greater in the Imagine condition (*M* = 5.18, *SE* = 0.20) as compared to the Story condition (*M* = 4.44, *SE* = 0.27), *b* = 0.58, *SE* = 0.12, *p* < .001. Crucially, this effect extended into actual donation behavior witnessed in the relief registry task: participants tended to donate more items that they had imagined themselves using to help others (*M* = 5.47, *SE* = 0.49) as compared to items seen in the Story condition (*M* = 4.63, *SE* = 0.50), *b* = 0.08, *SE* = 0.04, *p* = .024, Object condition (*M* = 3.73, *SE* = 0.51), *b* = 0.18, *SE* = 0.04, *p* < .001, or New condition (*M* = 4.40, *SE* = 0.44), *b* = 0.11, *SE* = 0.04, *p* = .004. A distinct manner of exploring whether prosocial intentions translated into prosocial behavior in this study would be to determine whether, on a trial-by-trial basis, higher willingness to help ratings predicted a greater likelihood of the item in question being donated in the relief registry task. To explore this, we conducted a mixed effects logistic regression (again, accounting for both by-stimulus and by-participant variance) with willingness to help as a predictor variable and item donations (1 = participant donated the item; 0 = participant did not donate the item) as an outcome variable but found this effect to be nonsignificant, *b* = 0.12, *SE* = 0.07, *p* = .086, OR *=* 1.13. Results pertaining to other measures (e.g., scene vividness) are provided in the S1 File.

## Study 2

### Method

#### Participants

A total of 35 participants were recruited in the same manner as in Study 1 and were compensated $25 for their participation. Five of these participants were excluded using the same comprehension checks and exclusion criteria as in Study 1, leaving a final sample of *N* = 30 individuals (age 18–27 years, *M* = 21.63 years, *SD* = 2.34, 12 males).

#### Procedure and materials

*Imagined helping (strong versus weak context) and control tasks*. The procedure of the imagined helping task used in Study 2 was the same as that used in Study 1 except for a few changes. First, participants now saw pairings of stories and items on all 30 trials as opposed to seeing story/image pairs on 20 trials and just images on the remaining 10 trials. Second, we used a different manipulation of task instructions than in Study 1. On 10 trials, participants were instructed to imagine themselves helping the person in the story using the donation item in the image at a location that is highly familiar to them (Imagine Strong Context condition). For example, if the location specified in the story was a playground, participants were told that they should imagine helping the person using the specified item at a playground that they had been to before and were familiar with. For the second set of 10 trials, participants received this same set of instructions but were asked to imagine a helping event taking place at an unfamiliar location (e.g., using the prior example, a playground that they had not been to before; Imagine Weak Context condition). The list of locations was taken from [70] and were randomized for each trial. On the remaining 10 trials, participants saw story/image pairs and were instructed to think about the writing style and likely source of the story/item pairing (Story + Object condition). This condition effectively collapsed the two control conditions from Study 1 into a single condition. The visual examples, practice trials, and timing for these tasks operated in the same manner as in Study 1. Randomization of order and the assignment of story/image pairs to condition also operated in the same manner as Study 1.

*Dependent measures*. Self-report. After completing all trials, participants saw the same 30 story/image pairs again and completed the same set of dependent measures as in Study 1 for each of these pairs.

In addition to these items, participants rated the familiarity of the location of the imagined helping event (location familiarity; 1 = not at all familiar to 7 = very familiar), whether they had been to that location before (1 = yes, 2 = no), and the extent to which they considered the person in need’s thoughts and feelings while imagining (theory of mind; 1 = not at all to 7 = strongly considered). Items related to imagined scenes (vividness, preliving, and location familiarity) were not completed for Story + Object condition as participants were not asked to imagine events on these trials. As in Study 1, scene detail and coherence were combined into a single vividness index (Cronbach’s *α* Imagine Strong Context = .94, Imagine Weak Context = .96). Following prior work on location familiarity [74], we eliminated trials on which participants failed to follow task instructions (i.e., Imagine Strong and Imagine Weak Context trials on which participants indicated that they had not been to or had been to the location imagined, respectively; average number of trials removed per participant: *M* = 1.00; *SD* = 1.78).

Relief registry. The same procedure for the relief registry as Study 1 was used with one exception: the endowment participants received to donate items was $10 instead of $20, forcing participants to prioritize some items over others (as they could only donate a maximum of 20 out of the 40 total items) and preventing them from simply donating one of every item as they were able to do in Study 1.

### Results

As in Study 1, we used generalized linear mixed-effects regression analysis to test for condition effects on our focal dependent measures while treating both participants and stimuli (i.e., story/image pairs, locations of imagined scenes) as random factors. We found an effect of imagined helping and strength of the visuospatial context on prosocial intentions: willingness to help was greater in the Imagine Strong Context condition (*M* = 5.08, *SE* = 0.20) as compared to both the Imagine Weak Context condition (*M* = 4.69, *SE* = 0.19), *b* = 0.37, *SE* = 0.13, *p* = .006, and the Story + Object condition (*M* = 4.35, *SE* = 0.22), *b* = 0.71, *SE* = 0.13, *p* < .001. Willingness to help was also greater in the Imagine Weak Context condition as compared to the Story + Object condition, *b* = 0.35, *SE* = 0.14, *p* = .012.

These effects extended into actual donation behavior during the registry task as in Study 1: participants donated more of the items presented in the Imagine Strong Context condition (*M* = 4.13, *SE* = 0.42) than in the Story + Object condition (*M* = 3.03, *SE* = 0.41), *b* = 0.14, *SE* = 0.04, *p* < .001, and the New condition (*M* = 2.83, *SE* = 0.27), *b* = 0.11, *SE* = 0.04, *p* = .003. Participants also donated more of the items presented in the Imagine Weak Context condition (*M* = 3.63, *SE* = 0.38) as compared to the Story + Object condition, *b* = 0.11, *SE* = 0.04, *p* = .005, and the New condition, *b* = 0.08, *SE* = 0.04, *p* = .041. The difference in donations between the Imagine Strong and Imagine Weak Context conditions was nonsignificant, however, *b* = 0.04, *SE* = 0.04, *p* = .316. As in Study 1, we conducted a mixed effects logistic regression to explore the relationship between trial-level willingness to help and item donations, finding this effect again to be nonsignificant, *b* = 0.08, *SE* = 0.05, *p* = .083, OR = 1.09. Results pertaining to other measures (e.g., scene vividness, theory of mind) are provided in the S1 File.

## General discussion

The current research introduces relief registries–online gift registries adapted in collaboration with charitable organizations–as a measure of prosocial behavior for use in psychological and behavioral economic research. Relief registries build upon prior work exploring real-world donation behavior using correlational [75] and field experimental methods [50–52], offering researchers an easy to use method for studying charitable donations in a way that is easily adaptable to a variety of research designs and strives to balance concerns about internal validity, ecological validity, and the ability to recruit large samples of participants in the lab or online.

To demonstrate this method, we explored whether a prior finding from our labs–that imagination can be leveraged as a tool to foster intentions to help others [20, 69–72]–would generalize to prosocial behavior in the form of actual donations of items participants had imagined using to help others. In other words, relief registries allowed us to directly probe whether the intention-behavior gap found in some moral psychological research [28–35] would reemerge in the context of a specific phenomenon from our own research. Consistent with previous work on imagination, we found effects of episodic simulation on both self-reported prosocial intentions as well as donation behaviors during the relief registry task; people who had imagined themselves using specific items to help targets in need expressed greater willingness to help those targets but were also more likely to actually give up their own money to donate those items in a subsequent task. Whereas willingness to help did not significantly predict donations on a trial-level basis, this may be due in part to the mismatch between the referent of the hypothetical and behavioral measures (i.e., the fact that the hypothetical measure referred to one’s willingness to help someone in an imagined scenario rather than willingness to donate items). Whereas we would once again remind readers not to overinterpret these effects due to the small sample sizes used here, we reinforce that these findings add to mounting evidence for a relationship between memory and imagination and prosocial intentions, emotions (e.g., empathy), and behavior [20, 69–72, 76–79].

Whether tradeoffs between internal and external validity are an inherent feature of research design in the social sciences has been the subject of some debate [80–83]. Although we by no means intend to resolve this dispute, relief registries allow for the direct observation of prosocial behavior in a setting which involves real stakes [6, 7] and has real-world impact all without sacrificing the level of internal validity required for causal inference. In this way, we see relief registries as complementary to related field experimental approaches to charitable giving from psychology and behavioral economics [e.g., 50–52] while being potentially more easily accessible and useful for different types of experimental designs than these existing approaches. For example, relief registries may be preferable over standard field experiments measuring monetary donations [e.g., 46, 65, 66] when researchers wish to allow participants to freely choose and donate items according to their own preferences (e.g., [84]) and may also work better than existing alternatives for certain types of research designs–for example, the studies reported above, which required participants to imagine themselves using specific items to help others before deciding whether they would like to donate these items. Relief registries are thus a useful addition to researchers’ methodological toolbox, particularly in light of recent debates about the extent to which self-reported attitudes and intentions predict actual behaviors [3, 4, 6, 7].

We acknowledge that relief registries still possess some of the limitations present in other lab-based work (e.g., possible demand characteristics or the fact that participants only just received the money they are donating, potentially leading to less careful decisions) [85] and this is one of the reasons we would stress that relief registries are intended to supplement rather than replace complementary approaches such as field experiments. Specific elements of relief registries could also be easily adapted to suit different researchers’ needs and address potential shortcomings in the way we utilized this method here. For example, we matched all donation items at the low price of $0.50 to avoid floor effects in giving and ensure that participants’ choices would not be influenced by differences in item costs; however, researchers may wish to use prices which better reflect the actual costs of donation items in order to heighten the mundane realism of this procedure and avoid the potential confound of participants’ choices being influenced by their perceptions of the value of the items (e.g., selecting more expensive items because it would benefit the charities more while costing the participant the same amount of money). At the same time, the bonus compensation provided to participants for use in the relief registry task itself ($20 and $10 in Studies 1 and 2, respectively) may be unrealistically high for researchers working with limited budgets, particularly those wishing to implement relief registries in larger samples of participants. However, prior research is optimistic with regard to the comparability of findings when using larger versus smaller-stakes donations [86, 87], suggesting that the validity of the relief registry approach would be maintained even when using smaller compensation amounts. Ultimately, though we have demonstrated the use of relief registries in just one format and one context here, we view the potential applications of this procedure as quite broad and encourage future researchers to explore ways to amend, adapt, and improve upon it in their own research.

The methods and results from our two experiments contribute to an important ongoing conversation about ecological validity in the fields of psychology and behavioral economics [3, 6–8, 11, 37], and moral psychology more specifically [9, 12, 31, 88]. As the field of psychology began to initially take shape in the early 20^th^ century, it was not uncommon for researchers to build new apparatuses for studying sensation, perception, and memory. Over a century later, it might behoove moral psychologists to return to our tinkering experimental roots and develop new tools for measuring and manipulating behavior, and in turn expand the ecological scope and ultimate impact of our lab-based work. Whereas relief registries are just one example of this process, we would argue that similar logic and strategies to those utilized here could be applied to the creation of other manipulations and measures in moral psychology and related fields. The theoretical rubber of moral psychology is beginning to hit the road of everyday life, and it seems to us an exciting frontier bridging traditional, in-lab manipulations with impact in the real-world lies ahead.

## Supporting information

S1 File (DOCX)
