# Supplementary material for: Moral psychology from the lab to the wild: Relief registries as a paradigm for studying real-world altruism
Source: PLoS One. 2022 Jun 13;17(6):e0269469. doi: 10.1371/journal.pone.0269469 (PMC9191725; doi:10.1371/journal.pone.0269469)
Supplement: S1 File — (DOCX) [file pone.0269469.s001.docx]

**Supplemental Materials**

**Role of Imagined Scene Vividness in Study 1**

The vividness of imagined helping episodes predicted willingness to help in the Imagine condition, *b* = 0.56, *SE* = .05, *p* < .001. Vividness also predicted the likelihood of donating an item in the Imagine and Object conditions, *b* = 0.04, *SE* = 0.02, *p* = .023.

**Roles of Imagined Scene Vividness and Theory of Mind (ToM) in Study 2**

The vividness of imagined scenes predicted participants’ willingness to help in the Imagine Strong Context condition, *b* = 0.58, *SE* = 0.06, *p* < .001, and Imagine Weak Context condition, *b* = 0.51, *SE* = 0.06, *p* < .001. Theory of mind also predicted willingness to help in the Imagine Strong Context condition, *b* = 0.50, *SE* = 0.05, *p* < .001, and Imagine Weak Context condition, *b* = 0.37, *SE* = 0.05, *p* < .001. Vividness was a nonsignificant predictor of donations in the Imagine Strong Context condition, *b* = 0.01, *SE* = 0.02, *p* = .564, and in the Imagine Weak Context condition, *b* = 0.02, *SE* = 0.02, *p* = .332. Theory of mind was a nonsignificant predictor of donations in the Imagine Strong Context condition, *b* = 0.00, *SE* = 0.02, *p* = .802, but significantly predicted donations in the Imagine Weak Context condition, *b* = 0.04, *SE* = 0.02, *p* = .011.
